# Genetically engineered mosquitoes, Zika and other arboviruses, community engagement, costs, and patents: Ethical issues

**DOI:** 10.1371/journal.pntd.0006501

**Published:** 2018-07-26

**Authors:** Zahra Meghani, Christophe Boëte

**Affiliations:** 1 Philosophy Department, University of Rhode Island, Kingston, Rhode Island, United States of America; 2 Institut des Sciences de l’Evolution de Montpellier, Université de Montpellier, CNRS, IRD, EPHE, Montpellier, France; Fundaçao Oswaldo Cruz, BRAZIL

## Introduction

Genetically engineered (GE) insects, such as the GE OX513A *Aedes aegypti* mosquitoes, have been designed to suppress their wild-type populations so as to reduce the transmission of vector-borne diseases in humans. Apart from the ecological and epidemiological uncertainties associated with this approach, such biotechnological approaches may be used by individual governments or the global community of nations to avoid addressing the underlying structural, systemic causes of those infections [1]. For instance, the rise in the number of Zika infections in northeastern Brazil is the product of the interaction of multiple factors. A key element is the 2015 El Niño climate phenomenon (in the context of global warming). A recent Human Rights Watch Report identified other factors responsible for the spread of the infection as the failure of the state to make adequate investment in piped water and waste services for the indigent segments of its population and to address racism and socioeconomic health disparities [1]. The report also criticized the violation of sexual and reproductive rights in Brazil. However, a detailed discussion of these structural, systemic factors lies beyond the scope of this Policy Platform, which is based on our expertise in healthcare ethics, political philosophy, feminist philosophy, medical entomology, insect–pathogen interactions, innovations in the control of vector-borne diseases, risk assessment, and environmental ethics.

We discuss here key ethical questions raised by the use of GE insects, with the aim of fostering discussion between the public, researchers, policy makers, healthcare organizations, and regulatory agencies at the local, national, and international levels. We affect that goal by outlining a procedural approach to decision-making about the use of the “biotechnology” that goes beyond “community engagement.” The protocol we advocate for entails informed deliberations and decision-making at the community level. It is designed to ensure that the voices of the marginalized and vulnerable groups that would be disproportionately affected by the decision are heard during the community-wide discussions. Moreover, we make the case that the values embedded in the risk assessment should be identified so that the community can make an informed decision about the use of GE insects. In addition, we advocate for the involvement of a variety of actors whose responsibility would be to ensure that the community has the opportunity to make an informed decision based on deliberations about the use of the “biotechnology.”

## GE insects: An emerging risk

The use of the GE insects in field trials or for the purposes of control of disease vectors qualifies as an emerging risk. The International Risk Governance Council has indeed defined “emerging risks” as those about which there is significant uncertainty and a low level of knowledge about the potential effect of and interactions with the system into which they are introduced [2]. Thus, rigorous risk assessment of the GE insect is crucial, as the affected community would need it to decide whether it would want the insect to be used in its neighborhood [3].

Released males GE OX513A (Box 1) are expected to out compete the wild males to mate successfully with their female wild-type counterpart. Of the resulting progeny, 96% are not expected to survive [4]. Consequently, after multiple releases over time, the population of the mosquito in the release area is expected to significantly decrease, which, presumably, will result in the decrease in the spread of the *Aedes*-transmitted arbovirus in humans. While the plan is to release billions of engineered mosquitoes [5] and given that (as of October 2017) at least 90 million GE OX513A *A*. *aegypti* mosquitoes have been released in various nations during field trials, and approximately 4% of that population of GE insect may have survived without tetracycline [4], it is worrisome that, as of yet, there are no published peer-reviewed studies about the fitness of that subpopulation of GE insects or its progeny. Moreover, as of yet, there are no peer-reviewed published studies about the epidemiological efficacy of this mosquito that demonstrate that field trials resulted in lower incidence of disease in humans.

**Box 1. The GE mosquito: How does it work?**

The GE OX513A *Aedes aegypti* has been subject to a germline modification that includes a lethality gene. Specifically, a synthetic genetic sequence encoding a tetracycline-repressible transcriptional activator (tTAV) is introduced into the mosquito with the intent of creating tetracycline dependency in the insect. In the absence of tetracycline, tTAV is expressed, and this leads to the death of most of the mosquitoes carrying the trait [4]. If tetracycline is present (as it is during the mosquito rearing in the laboratory, for example), then tTAV is repressed and the larvae can develop and reach adulthood. Female mosquitoes are the biters that spread the disease, so only the male GE mosquitoes are intended for release in the target area.

## Communities and informed decision-making

While it would be judicious to employ known-to-be-effective methods of disease vector controls [6], there is a push by some nonstate actors to develop and use GE insects [see, for instance, 7]. Nations with high-disease burden should be able to autonomously decide on the soundness of the “use” of GE insects, without undue influence from nonstate actors or other countries. They will shoulder the risks and harms from the use of GE insects. But the question of informed decision-making has relevance beyond the release area, as the *A*. *aegypti* mosquito has been inadvertently transported substantial distances in vehicles [8, 9]. Migration of GE mosquitoes over national borders could have serious international political implications.

Community advisory boards (CABs) have been proposed as a means for local community engagement [10]. In the context of international collaborative research in poorer nations, community engagement is praised as an indicator of sound ethical and research practice. However, there is a dearth of published research on models of effective engagement or methods for evaluating them [11, 12]. In fact, it appears that community engagement may have been used in some instances by developers of GE mosquitoes, working in conjunction with local government officials and agencies to ensure that affected populations perceive the “biotechnology” in a positive light and consent to its use [13]. In addition, the CAB model of community engagement may not necessarily be sensitive or responsive to power differential based on gender, race, ethnicity, age, nationality, class, or social status between CAB members, which might affect participants’ engagement in discussions [9] and, presumably, decision-making.

Given the limitations of CABs, in the interest of substantive community engagement, we propose a two-step approach to informed decision-making, which we term “Community Deliberations and Decision-Making” (CDD). Our approach recognizes that respect for the fundamental principle of democracy requires that affected parties be provided with the opportunity to make decisions about normative matters that impact them. The CDD protocol is also designed to be attentive and responsive to the power differential among groups within communities. So the aim of the CDD would be to provide the affected population with the opportunity to make an informed decision about whether they would want GE mosquitoes released in their neighborhood. But that would only be possible if government officials maintain a strict commitment to serve as the watchdogs of public health and the environment; they have a fiduciary duty to the people only. Thus, government agencies must subject claims by sponsors of new “products” to careful and rigorous scrutiny. (If government officials fail to be responsive to their duty to the public, in democracies, the people then have the ability to remove them from their office.) We also advocate for further research about the variety of complex normative elements that may come into play during field trials and open releases but which cannot be addressed in this Policy Platform.

As the first stage of the two-step CDD approach, the regional government, in conjunction with the relevant local grassroots nongovernmental organizations (NGOs) (for instance, organizations that advocate for women’s access to healthcare and which are unaffiliated with any entities involved in the development or sale of the GE insect), should invite representatives of the relevant populations constituting the community to participate in a series of forums about the use of the biotechnology. In the case of communities facing the threat of Zika, indigent females, specifically girls and women of childbearing age, are a significant relevant population, as they are the ones who could be infected with the virus during pregnancy [1]. Moreover, given traditional gender roles and inadequate social support, as mothers, they will bear the lion share of the enormous responsibility of caring and providing for the children harmed by the illness [14]. Given all that is at stake for indigent women of childbearing age, it would be critical to include them in the forums and for their stance to be given particular weight. Efforts should be made by the entities overseeing the forums (specifically, regional government entities, and local NGOs) to ensure that those women are afforded recognition in the forums as the equals of the other participants. Inequitable power relations between them and other groups in that community or nation must not be reinstantiated in the forums that are used to augment democratic processes.

The forum sessions should include consultations with experts in risk assessment and insect vectors of disease who are unaffiliated with the developers of the GE mosquito. We have two worries about the presence of specialists associated with the developers of the GE insect at the forum consultations. First, their presence might inhibit participants from openly articulating their concerns. Second, the experts affiliated with the organizations that sponsored the GE insect might present themselves to the forum participants as having greater epistemic authority than the unaffiliated experts, undermining the latter’s epistemic credibility and thus having an undue influence over the deliberations and decision-making. Thus, we propose that GE mosquito developers share all their data in a transparent manner as requested by the independent experts and answer any questions they might have about their research if they are called on by them. The cost of the forums’ consultation with the unaffiliated experts should be borne by the sponsoring entity of the GE insect.

The onus would be on the independent specialists to evaluate the risk assessments provided by the sponsor of the GE mosquito and explain its significance to the forum members (which, presumably, would be constituted of laypersons). The values that shaped the risk evaluation, ranging from hazard identification to dose-response modeling to exposure assessment to risk characterization [15] would have to be identified. That would enable the members of the forum to make an informed decision about the use of the GE mosquito. For instance, is the risk evaluation predicated on the normative assumption that it is acceptable that a percentage of GE mosquitoes (and their progeny) might survive and establish a GE population in the local ecosystem even though the long-term impact of that possibility on the ecosystem is not known? Does the risk assessment favor the use of the new “biotechnology” whose ultimate effectiveness remains unproven even though evidence-based, effective, sustainable, and cheaper approaches are available?

The second step in the informed decision-making process would involve community wide discussions and decision-making [16, 17]. (The forums are proposed as a tool for augmenting democratic processes to ensure that marginalized and vulnerable groups that might be disproportionately affected by the decision about the use of a GE mosquito are not drowned out in democratic processes wherein majoritarianism prevails; their rationale and decision would be shared with the larger community as it deliberated on the use of the “biotechnology” and it must take them into account as it makes its decision.)

The community level decision-making would have to be prefaced by an iterative deliberative process wherein members of the community along with scientists, public health experts, policy makers, and regulatory agency personnel would identify their normative concerns and justify their stance to each other (see [17] on democratic engagement). The sessions would provide participants with the opportunity to engage with each other by framing and reframing ethical and sociopolitical issues. They would also have “space” to articulate and examine the new normative concerns and questions that might emerge.

We want to acknowledge that the community members would hear the independent scientific experts’ analysis of the risk assessment of the use of the GE mosquito (relative to that of other approaches to controlling the spread of arboviruses or their vectors) in an emotionally and normatively charged context (specifically, they may be fearful about the spread of the arboviruses or their emergence, and they may subscribe to particular religious or cultural values). However, since the community will be affected by the choice to use or not use the GE mosquito, it must make that decision.

We also want to recognize that the affected community may include a diversity of standpoints and interests. However, we believe it is possible for community members to deliberate and decide about matters of common concern. After all, democratic deliberations and decision-making occur at many levels and in many nations even though the participants have varied interests, values, and political agendas. The key issue is that the process of deliberations and decision-making should be fair, and the results should not reinstantiate or create inequitable power relations among the groups within the community.

With respect to GE animals with gene drives, their release in the wild should necessarily be preceded by global deliberations and decision-making. Depending on its genetic characteristics, a gene drive cassette could spread in an entire population and affect it within a fairly short timespan [18] leading to unwanted repercussions across ecosystems on multiple species whose “ways of life” [19] are mutually entangled [20].

## Cost of the patented GE insect

The cost of the “biotechnology” is another key ethical issue. Initially, the Oxitec GE mosquito had been classified by the United States Food and Drug Administration (FDA) as a pharmaceutical product, albeit a new animal drug. But in October 2017, the agency handed regulatory authority to the US Environmental Protection Agency (EPA), which intends to regulate the insect as a “pesticide.” The transfer of regulatory jurisdiction was justified on the grounds that the GE mosquito had been classified as a pesticide by Oxitec. In October 2017, the FDA issued a guidance document for industry that the EPA would have jurisdiction over mosquitoes categorized as “pesticides” and the FDA would regulate GE insects that were construed as ‘products’ that were “intended to prevent mosquito-borne disease in humans or animals.” However, the change in regulatory jurisdiction and the classification of the GE mosquito as a pesticide does not change the fact that it is meant to reduce or prevent the transmission of arboviruses in humans, and thus, it is a public health “tool.” That raises the following question for poorer nations: How much will the GE insects cost and how will local or federal governments pay for them? The cost is likely to be significant for those nations and will eat into their budgets (Box 2).

**Box 2. GE mosquitoes in Piracicaba**

The Oxitec GE mosquito is projected to cost the Brazilian city of Piracicaba (with a population of 391,449) approximately US$1.1 million over a two-year period at the cost of US$10 per person in the target area (50% of the cost will be financed by the city’s current mosquito control budget; Oxitec will be subsidizing some portion of the cost, presumably, to build a market for its “product”) [4]. The use of the GE mosquito will require recurrent relicensing from the patent owning company [5]. However, this estimate seems relatively low compared to a recent one that suggests that the cost of using the Oxitec GE mosquito for an urban population of 50,000 would be approximately US$1.9 million in the first year and US$384,000 each year thereafter [21]. While different situations and contexts may naturally lead to different costs of intervention, the discrepancy is troubling, given the importance of reliable and accurate financial information for informed discussions and choices by concerned communities and public health authorities, especially in countries that have stringent limits on their public health budgets.

The use of the GE insect raises another related complex ethical, socio-political and legal issue complicating the biological ones. At least 4% of the GE insects will survive even in absence of tetracycline. If they establish outside of the target area, then questions must be asked about the extent of the patent holders’ power. To appreciate how the judicial systems of various nations may treat such cases it might be useful to consider the (partially) analogous case of patented GM (genetically modified) seed (Box 3).

**Box 3. Patents and GE organisms**

In Canada and the US, the courts have invariably sided with the patent holder in the case of genetic drift (i.e., the migration of GM seed into farmland meant to grow non-GM plants) [22]. In Monsanto Canada Inc. versus Schmeiser, the Canadian Supreme Court found farmer Percy Schmeiser guilty of patent infringement, even though there was evidence that he had not intentionally planted GE corn on his land and that its presence on his farm was inadvertent; the wind had blown it onto his land, and it had taken root. Wilson has noted that “armed with the court-enforced strength of its patents, Monsanto aggressively seeks out any growers that may either intentionally or unintentionally infringe upon those patents” [23].

The US and Canadian courts could consider the rulings in the genetic drift cases as the relevant precedent. So if a habitat of GE insects is found in a nontarget area, then the courts could accept the patent-holding corporation’s argument that the owner of that property was “using” its “product” without paying for it. With respect to other nations, given that one of the key aims of the World Trade Organization (WTO) is to protect the rights of patent-holding entities, it may rule in favor of them in disputes involving inadvertent “use” of the GE insects by a WTO member nation. That kind of “use” is likely if the releases are in border regions because borders of nations arbitrarily carve up ecosystems.

The enforcement of the right of patent-holding entities in cases where the affected nations have not consented to the “use” of the “pesticide” presents a thorny political and economic problem for poorer nations. Patent-holding corporations could demand additional fees from those countries, claiming that they are “using” its product. Given that their budgets are already strained, those costs could further undermine those nations’ capacity to provide basic necessities for their population [24]. They may even have to take out additional loans from transnational financial institutions to pay those costs, worsening their national debt burden.

## Conclusion

We have identified the complexity of some of the moral issues and the need for concerted, comprehensive local, national, and global efforts to find effective, efficient, and ethical solutions for reducing the transmission of arboviruses. We have focused primarily on the use of the GE mosquito OX513A, but our analysis is relevant for the larger category of gene drive-based approaches for insects and other animals.

## References

[1] Human Rights Watch. Neglected and Unprotected: The Impact of the Zika Outbreak on Women and Girls in Northeastern Brazil. 12 July 2017. https://www.hrw.org/report/2017/07/12/neglected-and-unprotected/impact-zika-outbreak-women-and-girls-northeastern-brazil

[2] International Risk Governance Council. Governance of Emerging Risks. 2017. https://www.irgc.org/risk-governance/emerging-risk/

[3] Meghani Z, Kuzma J. Regulating animals with gene drive systems: lessons from the regulatory assessment of a genetically engineered mosquito. Journal of Responsible Innovation 5, no. sup1 (2018): S203–S222. 10.1080/23299460.2017.1407912

[4] Phuc HK, Andreasen MH, Burton RS, Vass C, Epton MJ, Pape G, et al Late-acting dominant lethal genetic systems and mosquito control. BMC Biol. 2007; 5:11 10.1186/1741-7007-5-11 17374148 PMC1865532

[5] Servick, K. Brazil will release billions of lab-grown mosquitoes to combat infectious disease. Will it work? 2016. Sciencemag.org.

[6] Boëte C, Reeves RG. Alternative vector control methods to manage the Zika virus outbreak: more haste, less speed. The Lancet Global Health, 2016 4, 6, e363 10.1016/S2214-109X(16)00084-X 27198836

[7] Regalado A. Bill Gates Doubles His Bet on Wiping Out Mosquitoes with Gene Editing. MIT Technology Review. 2016. https://www.technologyreview.com/s/602304/bill-gates-doubles-his-bet-on-wiping-out-mosquitoes-with-gene-editing/

[8] Oxitec. Draft Environmental Assessment for Investigational Use of Aedes aegypti OX513A. http://www.fda.gov/AnimalVeterinary/DevelopmentApprovalProcess/GeneticEngineering/GeneticallyEngineeredAnimals/ucm446529.htm, p.66

[9] Gloria-Soria A, Brown JE, Kramer V, Hardstone Yoshimizu M, Powell J R. Origin of the dengue fever mosquito, *Aedes aegypti*, in California. PLoS Negl Trop Dis 2014; 8, 7: e3029 10.1371/journal.pntd.0003029 25077804 PMC4117443

[10] Neuhaus CP, Caplan AL. Ethical lessons from a tale of two genetically modified insects. Nat Biotechnol. 2017; 35(8):713–6. 10.1038/nbt.3927 28787406

[11] Maung Lwin K, Cheah PY, Cheah PK, White NJ, Day NP, Nosten F, et al Motivations and perceptions of community advisory boards in the ethics of medical research: the case of the Thai-Myanmar border. BMC Med Ethics. 2014; 15:12 10.1186/1472-6939-15-12 24533875 PMC3929312

[12] Kolopack PA, Parson JA, Lavery JA. What makes community engagement effective?: Lessons from the Eliminate Dengue Program in Queensland Australia. PLoS Negl Trop Dis 2015; 9, 4: e0003713 10.1371/journal.pntd.0003713 25875485 PMC4395388

[13] de Campos AS, Hartley S, de Koning C, Lezaun J, Velho L. Responsible Innovation and political accountability: genetically modified mosquitoes in Brazil. *Journal of Responsible Innovation*. 2017: 4(1) 5–23-19. 10.1080/23299460.2017.1326257

[14] Diniz D. Zika virus, women and ethics. Dev. World Bioeth. 2016; 16 (2): 62–3. 10.1111/dewb.12119 27378033

[15] Meghani Z. Regulation of Consumer Products, *Consumer Perception of Product Risks and Benefits* eds. Emilien G., Weitkunat R. and Luedicke F. (2017). Springer

[16] Macer D. Ethics and community engagement for GM insect vector release In: Boëte C, editor. Genetically Modified Mosquitoes for Malaria Control. Georgetown, Texas: Landes Biosciences, Eurekah; 2006.

[17] Benhabib S. Strong Democracy and Transnational Legal Spheres: Are They Compatible? In: Norris T, editor. In Strong democracy in crisis: Promise or peril?: Lexington Books; 2016 pp. 79–92.

[18] Burt A. Site-specific selfish genes as tools for the control and genetic engineering of natural populations. Proc Biol Sci. 2003; 270 (1518): 921–8. 10.1098/rspb.2002.2319 12803906 PMC1691325

[19] Rose DB. Val Plumwood’s Philosophical Animism: attentive interactions in the sentient world. Environmental Humanities. 2013; 31(1):93–109. 10.1215/22011919-3611248

[20] Courtier-Orgogozo V, Morizot B, Boëte C. Agricultural pest control with CRISPR-based gene drive: time for public debate: Should we use gene drive for pest control? EMBO Rep. 2017; 18(6): 878–80. 10.15252/embr.201744205 28512121 PMC5452019

[21] Alfaro-Murillo JA, Parpia AS, Fitzpatrick MC, Tamagnan JA, Medlock J, Ndeffo-bah ML, et al A Cost-Effectiveness Tool for Informing policies on Zika Virus Control. PLoS Negl Trop Dis 2016 10(5):e0004743 10.1371/journal.pntd.0004743 27205899 PMC4874682

[22] Pechlaner G, Otero G. The neoliberal food regime: Neoregulation and the new division of labor in North America. Rural sociology. 2010; 75(2):179–208. 10.1111/j.1549-0831.2009.00006.x

[23] Wilson S. Induced Nuisance: Holding Patient Owners Liable for GMO Cross Contamination. Emory Law Journal. 2014 64: 169–198.

[24] Gill S, & Benatar SR. History, Structure and Agency in Global Health Governance: Comment on “Global Health Governance Challenges 2016 –Are We Ready?” International Journal of Health Policy and Management. 2017 6(4), 237–241. 10.15171/ijhpm.2016.119 28812808 PMC5384987

